# General Self-Efficacy Among Pregnant Women Attending Antenatal Care Units in Tunisia and Its Association with Family Quality of Life: A Multicenter Cross-Sectional Study

**DOI:** 10.3390/healthcare13233069

**Published:** 2025-11-26

**Authors:** Maha Dardouri, Fatma Korbi, Hajer I. Motakef, Hamdi Lamine, Shaima Mohammed Nageeb, Bushra Alshammari, Sihem Chahed, Martin Rusnák, Imen Ayouni

**Affiliations:** 1 Community Health Nursing Department, College of Nursing, University of Hail, Hail 21424, Saudi Arabia; m.dardouri@uoh.edu.sa (M.D.); h.lamine@uoh.edu.sa (H.L.); 2 Institut Supérieur Privé des Sciences de la Santé, Upsat Sousse, Sousse 4000, Tunisia; fattmakorbi@gmail.com; 3 Maternal and Child Health Nursing Department, College of Nursing, University of Hail, Hail 21424, Saudi Arabia; ha.matekef@uoh.edu.sa; 4 Mental Health Nursing Department, College of Nursing, University of Hail, Hail 21424, Saudi Arabia; sm.nageeb@uoh.edu.sa; 5 Medical Surgical Nursing Department, College of Nursing, University of Hail, Hail 21424, Saudi Arabia; bu.alshammari@uoh.edu.sa; 6 Midwifery Department, Higher School of Health Sciences, University of Monastir, Monastir 5000, Tunisia; chahedsihem340@gmail.com; 7 Department of Public Health, Faculty of Health Care and Social Work, Trnava University, 918 43 Trnava, Slovakia; martin.rusnak@truni.sk; 8 Department of Epidemiology, University Hospital Farhat Hached, Sousse 4000, Tunisia

**Keywords:** self-efficacy, family quality of life, pregnant people, perinatal care, midwifery

## Abstract

**Highlights:**

**What are the main findings?**

- The prevalence of low general self-efficacy (GSE) among pregnant women was notably high, particularly among those with at least one child.
- Age, rural area, planned pregnancy, family quality of life, parenting, and family material well-being were predictors of GSE among pregnant women.

**What are the implications of the main findings?**

- Investment in multidisciplinary, family-centered care teams is essential to address the complex psychosocial needs of expectant families.
- Midwifery-led interventions should be designed to assess and respond to family and community-level determinants, fostering a shared understanding of pregnancy and parenting between partners.

**Abstract:**

**Background/Objectives:** General-self efficacy (GSE) is a substantial element during pregnancy that promotes healthy decision-making and prevents complications. Information on predictive factors of GSE among pregnant women is limited. This study aimed to assess the GSE among pregnant women and identify its relationship with family quality of life (FQOL) domains in a lower-middle-income community. **Methods**: This cross-sectional analytical study was conducted in nine antenatal care centers from July 2024 to March 2025. Pregnant women were enrolled through the multiple stage sampling method. GSE in pregnant women was assessed using the General Self-efficacy Scale. FQOL was assessed using the Beach Center Family Quality of Life Scale. Univariable and multivariable linear regression analyses were performed to assess predictors of GSE among pregnant women. **Results**: A total of 417 pregnant women participated in the study. The prevalence of low GSE was 12.2%. Multivariable linear regression showed that older age (*p* = 0.02), rural area (*p* = 0.007), and planned pregnancy (*p* = 0.03) were predictors of GSE among pregnant women. The total score of FQOL (*p* = 0.0001), parenting (*p* = 0.004), and material well-being (*p* = 0.043) were positive determinant factors of GSE in pregnant women who have at least one child. **Conclusions**: The prevalence of low general self-efficacy (GSE) among pregnant women was notably high, particularly among those with at least one child. Education regarding family planning, parenting, and financial management through multidisciplinary, family-centered care teams is essential to address the complex needs of expectant families.

## 1. Introduction

According to the World Health Organization, 700 women died worldwide in 2023 from modifiable risk factors related to pregnancy and childbirth [1]. Almost 92% of all maternal deaths occurred in low- and lower-middle-income countries due to preventable complications before and during pregnancy [1]. Indeed, quality of care is the most well-known factor that leads to complications of pregnancy [2]. Midwifery care plans that employ adherence to healthy behaviors were implemented to improve the quality of care for pregnant women [3]. In Tunisia, midwives play a crucial role in maternal and reproductive health during the pregnancy, childbirth, and postpartum periods. They are increasingly involved in maternal health promotion and education aiming at maternal adherence to healthy behaviors [4,5].

Evidence showed that successful behavior changes are predicted by the individual’s self-efficacy [6,7]. According to the Social Cognitive Theory (Bandura, 1997), perceived self-efficacy refers to the belief that one can perform novel or difficult tasks and achieve desired outcomes [8]. Pregnancy is a rich interplay of physical changes, emotional adjustment, and familial adaptations [9,10,11]. Pregnant women’s general self-efficacy (GSE) is important in recognizing and coping with life changes related to pregnancy and childbirth. Previous research revealed that perceived GSE in pregnant women is related to their self-abilities in terms of health practices and their health status management [12,13]. Another study showed that GSE reduces symptoms of anxiety in pregnant women [14]. Therefore, it is crucial to put an emphasis on GSE during pregnancy and its associated factors. This will help midwives to settle tailored-healthcare plans that promote healthy pregnancies, improve the quality of care, and thus reduce maternal morbidity in vulnerable regions.

Contemporary research has supported the family-oriented care approach, as the patient’s family is a core dimension and a natural support system that can promote both individual and family health [15]. The recent literature reported a close correlation between family health and self-efficacy in different populations [16]. For instance, a study showed that good family support significantly affected patient recovery, self-care behavior, and self-efficacy among patients with diabetes mellitus [17]. Other studies revealed that individuals with larger family support networks are more likely to have higher self-efficacy [18,19,20]. Puspasari et al. showed that family functioning can improve maternal self-efficacy among adolescent mothers [21]. These studies shed light on a potential GSE source, which is family health. Family quality of life (FQOL) is a multidimensional concept that is largely used as an important outcome of family health [22]. Poston et al. defined four functioning domains of the FQOL which are daily family life, parenting, family interactions, and financial well-being [23]. Other researchers added other dimensions to FQOL such as family relationships and family well-being [24].

Recently, researchers underlined the literature gap existing in the examination of the association between pregnant women’s self-efficacy and family functions, underscoring the importance of family context in this topic [19,25]. In Tunisia, extended families remain as important as nuclear families. They are potentially influential in the reproductive life of couples [26]. The authors believe that understanding the association between GSE among pregnant women and FQOL in a family-friendly context can renovate midwifery interventions regarding maternal health education and promotion. Therefore, this study aimed to assess the GSE among pregnant women attending antenatal care units in Tunisia and identify the relationship between GSE and FQOL domains.

## 2. Materials and Methods

### 2.1. Study Design and Settings

This was a multicenter cross-sectional study that was conducted in nine antenatal care clinics in the city of Sousse, located in the eastern center of Tunisia, from July 2024 to March 2025. The clinics are public sector facilities that provide antenatal care. In Tunisia, the public health sector contains three levels of care: primary care centers and district centers, regional hospitals, and university hospitals. In this study, all levels are targeted at clinics in the center-east (Sousse region) that provide care for a wide range of populations from all over the country [27].

### 2.2. Population, Sampling, and Bias

The target population is pregnant women aged 18 to 45 years from all socioeconomic groups, regardless of pregnancy risk status. Multi-stage sampling techniques were employed. In stage 1, researchers randomly selected healthcare centers from a list of 16 centers providing antenatal care, located in the region of the study, using Excel. The list contains different types of clinics, including a mother and child healthcare center, primary healthcare centers, regional hospitals, and university hospitals. These settings provide antenatal care to a heterogeneous population. In the second stage, the researchers simply selected random days for data collection. On the selected days, all participants are included, given the low daily participant numbers (5 to 10).

### 2.3. Sample Size Calculation

The sample size was calculated using the single population proportion formula. Since the prevalence of low GSE was unknown and no prior research had been conducted in countries with similar socioeconomic characteristics, this study used an estimate of 50%, with a 5% margin of error and a 95% confidence level. The minimal sample size was 385 [28].

### 2.4. Variables and Data Collection Instruments

To reduce information bias, the authors used valid and reliable instruments. General self-efficacy (GSE) was measured using the General Self-efficacy Scale (GSES) validated among Arab women [29,30]. The GSES assesses the strength of an individual’s belief in their own ability to respond to novel or difficult situations and to deal with any associated obstacles [30]. This scale is composed of 10 items scored on a four-point Likert scale (from 1, “not at all true”, to 4, “Exactly true”). The total general self-efficacy score is calculated by selected points of items, giving a maximum total score of 40. A higher score indicates higher GSE. GSE was classified according to the total score as follows: ≤20 indicates low GSE, 21 to 30 indicates moderate GSE, and 31 to 40 indicates high GSE [31]. The high reliability, stability, and construct validity of the GSES have been confirmed in previous studies among pregnant women [32,33,34]. The GSES’s reliability, measured using Cronbach’s alpha, is 0.85, with internal consistency ranging from 0.76 to 0.91 among pregnant women [32].

According to a scoping review, the most used and effective scales for assessing the FQOL are the Beach Center Family Quality of Life Scale [35] and the Family Quality of Life Survey-2006 [36]. These instruments are applicable for families including or not including a family member with disabilities [37]. In this study, FQOL was assessed using the “Beach center family quality of life scale” due to the availability of an Arabic version (BCFQOL-AR) [38]. This instrument is one of the most used in literature in FQOL assessments due to its validity and reliability [24,35,37,38]. It is composed of 25 items divided into five dimensions for families of children with disabilities. For families of children without disabilities, only the first 21 items are used for FQOL assessment [39,40]. In this study, only pregnant women who have healthy children filled in this scale. Items were grouped into four dimensions (D1: family interaction: items 1–6; D2: parenting: items 7–12; D3: emotional well-being: items 13–16; D4: material well-being: items 17–21). The participant indicates on a five-point Likert scale her level of satisfaction with each item (1: not at all satisfied; 5: very satisfied). The calculation of the scores for each dimension is performed by adding the points (1 to 5) provided by the participants. Higher scores indicated a higher level of satisfaction with FQOL domains. Studies have shown strong internal consistency for the overall scale and its subscales, along with evidence of construct validity, supporting its use in measuring family quality of life in Arabic-speaking contexts [37,38]. The BCFQOL-AR demonstrates strong internal consistency, with overall scale reliability typically above 0.95 and subscale reliability ranging from 0.85 to 0.95. This indicates that the items within each subscale are measuring the same underlying construct and that the scale is consistent in its measurement [24,38].

Sociodemographic and general characteristics were recorded, including age, place of residence, marital status, level of education, number of family members, monthly income, household income level, occupation, number of children, and children under 5 years. Clinical and obstetrical information included gestational age, para, gravida, pregnancy complications such as gestational diabetes mellitus or pre-eclampsia, planning of pregnancy, smoking, and receiving general support from the husband or not (physical and moral).

### 2.5. Data Analysis

Data entry, descriptive analysis, and score calculation were performed by IBM SPSS Statistics 20.0. R statistics software (version 4.3.1) was used to perform univariable and multivariable linear regression analyses. Quantitative variables were expressed as mean and standard deviation (SD). Qualitative variables were expressed in frequencies and percentages. Normal distribution of continuous variables was verified using the Kolmogorov–Smirnov test. To determine predictors of GSE and assess the relationship between GSE and the family quality of life, the authors performed univariable and multivariable linear regression analysis. Since the FQOL instrument was not applicable for families who did not have children, results were presented for all samples and for the subgroup of pregnant women who have at least one child. To reduce the collinearity effect related to the FQOL total score and domains, two different linear regression models were used. The first model used the four dimensions of the BCFQOL (family interaction, parenting, emotional well-being, and physical/material well-being) as exposures. The second model used only the FQOL total score as an exposure. The significance level was defined as *p* < 0.05 with a confidence interval of 95%.

### 2.6. Ethical Considerations

Ethical approval was obtained from the Human Research Ethics Committee at Farhat Hached University Hospital, Sousse, under the reference number IORG 0007439 ERC 02092024. The participants were informed about the purpose of the study, the voluntary participation, and the right to drop out. Written informed consent was obtained from all participants, and the collected data were coded and treated anonymously and confidentially.

## 3. Results

### 3.1. Sociodemographic and Clinical Factors

In this study, 417 pregnant women were enrolled. Table 1 shows that the mean age of participants was 30.24 ± 5.4 years, and they were living mostly in urban areas (79.9%). Most of the participants had university diplomas (42.9%), employment (55.4%), and a monthly income equal to three to four times the minimum wage in Tunisia (42.2%). Almost seventy-seven percent of the participants perceived that their socioeconomic level was moderate (n = 319). More than half of the pregnant women declared that they received general support from their husbands (65.9%).

Table 2 presents the obstetrical and clinical information of the participants. Most of the women (82.9%) were in the second or third trimester of pregnancy. More than half of the subjects had two or more pregnancies (66.9%), one or more parities (62.8%), no current pregnancy complications (64.5%), no previous abortion (68.1%), and no previous C-section (69.3%).

### 3.2. General Self-Efficacy and Family Quality of Life

Table 3 describes the sample in terms of GSE and FQOL domains. Almost half of the subjects reported moderate GSE with a mean total score of 28.15 ± 6.016. The means of the family interaction and parenting scores among pregnant women who have at least one child were, respectively, 25.06 ± 4.659 and 24.65 ± 4.849, from a total domain score of 30. The mean of material well-being was greater than the mean of emotional well-being.

Table 4 presents the findings of the linear regression analysis performed for the whole sample to assess the relationship between general self-efficacy and the characteristics of pregnant women attending antenatal care units. Multivariable analysis showed that age (*p* = 0.02) and rural area (*p* = 0.007) negatively determined the GSE of pregnant women. Planned pregnancy was positively determined by GSE (*p* = 0.030). Univariable analysis revealed that unemployment (*p* = 0.033), low household income (*p* < 0.001), and complications during pregnancy (*p* = 0.028) were negatively associated with GSE. However, high household income was positively related to GSE (*p* = 0.044).

For pregnant women who have children, univariable analysis shows that low household income level (*p* = 0.001) and rural area of residence (*p* = 0.002) were negatively associated with GSE (Table 5). All FQOL domains were positively associated with GSE (*p* < 0.001). Multivariable analysis revealed that the total scores of FQOL (*p* < 0.001), parenting (*p* = 0.004), and material well-being (*p* = 0.043) were positive determinant factors of GSE in pregnant women who have at least one child.

## 4. Discussion

### 4.1. GSE in Pregnant Women

In the context of pregnancy, GSE is considered an important psychological resource as women face numerous physiological, emotional, and social adjustments. GSE empowers women to effectively cope with the physical and emotional demands of pregnancy, enhancing their ability to make informed health decisions, manage stress, and maintain psychological resilience [7,41]. Previous research revealed that GSE mediates the association between family functions and depressive symptoms among pregnant women. The current findings revealed that twelve in one hundred pregnant women had low GSE and half of the sample showed moderate GSE. Higher prevalence of low GSE was registered in China (22.6%) and Indonesia (19.2%) [13,25]. This could be explained by misreporting on subjects regarding their real GSE status.

### 4.2. GSE and FQOL

In our knowledge, this was the first study to assess FQOL during the perinatal period and its association with GSE among pregnant women, particularly those who have at least one child. FQOL, parenting, and physical/material well-being positively correlated with GSE in pregnant women who had at least one child. This means that pregnant women with at least one child who were satisfied with their FQOL, their parenting style (child rearing), and their physical/material well-being were likely to have higher GSE levels. Conversely, pregnant women with at least one child who were not satisfied with their FQOL, their parenting style (child rearing), and their physical/material well-being were likely to have lower GSE levels.

Family interaction and emotional well-being were not predictors of GSE, which is unconcordant with the results of previous research [25]. In another context, lower family functions, including family emotional connection and communication, decreased GSE among pregnant women. These differences could be explained by socioeconomic disparities and fewer job opportunities in lower-middle-income countries. Indeed, in this study almost half of the sample were unemployed and 11.3% had a family monthly income lower than the minimum wage in Tunisia [42]. This must create substantial physical/material issues within the family. Consequently, the GSE of pregnant women depends on their satisfaction with their physical/material well-being, especially in pregnant women with children. In a similar context, researchers found that being a career person (employment) was a predictor of good GSE among pregnant women in Indonesia [13].

### 4.3. GSE and General Factors

Simon et al. found that low childbirth self-efficacy was related to unplanned pregnancy and age ≤ 24 years [43]. This was similar to the current findings. Among all the pregnant women, age and planned pregnancy positively correlated with GSE. This means that older women and women who planned their current pregnancies had a higher GSE level. Another study found that fertile age was a predictor of good GSE in comparison to high-risk age (35 years) among pregnant women in Indonesia [13].

This study revealed that rural places of residence negatively correlated with GSE. This means that women who are resident in rural areas are more likely to have lower GSE. In fact, health inequalities in Tunisia represent a significant challenge, driven primarily by stark geographic disparities between rural and urban regions in terms of access to healthcare services [44]. The public health sector in Tunisia comprises three lines of care: the first line consists of basic health groups, basic health centers, and district hospitals; the second line comprises regional hospitals, generally located in the main towns of the governorates; and the third line involves university hospitals and specialist centers [45]. While the public healthcare system in Tunisia provides a wide range of health services including preventive care, curative care, prenatal care, family planning, and promotional services, as well as maternal and child protection, women living in rural areas usually have limited access to these services, especially in the second and the third lines [44,45].

Also, this association could be indirectly related to educational level, considering that more than half of the subjects did not have an academic education. In Tunisia, rural women usually had limited access to higher education due to cultural limitations and distance [46,47]. Previous research found that having higher education was a predictor of good GSE [13].

### 4.4. Limitations

This study presents some limitations. First, cross-sectional design is usually associated with an information bias related to self-reported data. Second, the limited number of family assessment tools validated in the Arabic language restricted the researchers’ choice [37,38]. Although BCFQL-AR is reputable in assessing the FQOL of children with disabilities, it was also used for families with healthy children [39,40]. Third, in cross-sectional studies reverse causality cannot be excluded, which is true in the present case. Nevertheless, this study used a validated and reliable scale to assess GSE and a random sampling method with a representative sample size recruited from multiple centers. These facts produced robust results that can be generalizable. Despite the crucial role GSE plays in managing a healthy perinatal period, most previous research [43,48] has focused on childbirth self-efficacy. This fact produces a limitation in discussing the present results. The current findings regarding the prevalence of GSE and its relationship with women’s characteristics can be generalizable to all pregnant women in Tunisia. However, given the sample size of pregnant women who have at least one child, the results regarding the association between GSE and FQOL cannot be generalizable.

### 4.5. Implications for Practice

This study highlights the importance of maternal health in lower-middle-income countries, focusing on Tunisia. It emphasizes that GSE is a modifiable factor and that midwives play a pivotal role in strengthening it through holistic and family-oriented care. Enhancing GSE and family health can reduce maternal stress and improve postpartum mental health [49].

This research calls for expanding the role of midwives beyond clinical duties to include family education, psychosocial support, and active listening during antenatal care. Integrating GSE screening into care plans and fostering family-centered approaches are key recommendations. It also suggests that public health policies should fund multidisciplinary, family-focused teams and promote partnerships with social services to better address families’ material, educational, and emotional needs.

Finally, this study recommends conducting randomized controlled trials to evaluate the effectiveness of prenatal family-oriented programs and to explore how family quality of life (FQOL) influences GSE among pregnant women with children.

## 5. Conclusions

Low GSE was found to be common among pregnant women, especially those with children. Lower GSE levels were linked to dissatisfaction with FQOL, parenting style, and material well-being. This study emphasizes the need for family-oriented midwifery care that addresses family needs and promotes shared understanding between partners. Midwife-led interventions, developed collaboratively with social welfare services, should support families to improve FQOL. The findings also underline the importance of future research to examine the causal link between FQOL and GSE through multidisciplinary, family-centered programs.

## Figures and Tables

**Table 1:** Sociodemographic data of participants (n = 417).

Variables		n (%)
Area of residence	Urban	333 (79.9)
	Rural	84 (20.1)
Level of education	Illiterate	19 (4.6)
	Primary	55 (13.2)
	Secondary	164 (39.3)
	University	179 (42.9)
Employment status	Employed	231 (55.4)
	Unemployed	186 (44.6)
Family monthly income	TND < 500 *	47 (11.3)
	TND 500–999	138 (33.1)
	TND 1000–2000	176 (42.2)
	TND >2000	56 (13.4)
Household income level	Low	75 (18)
	Moderate	319 (76.5)
	High	23 (5.5)
Marital status	Married	397 (95.2)
	Divorced	18 (4.3)
	Widowed	2 (0.5)
Age (m ± SD)		30.24 ± 5.414
Household size (m ± SD)		3.20 ± 1.21

* TND: Tunisian dinar.

**Table 2:** Clinical and obstetrical data of participants (n = 417).

Variables		n (%)
Gestational age	First trimester	71 (17)
	Second trimester	174 (41.7)
	Third trimester	172 (41.2)
Gravida	One	138 (33.1)
	Two or more	279 (66.9)
Parity	Nulliparous	155 (37.2)
	Multiparous	262 (62.8)
Number of live births	None	155 (37.2)
	One or more	262 (62.8)
Current pregnancy complications	No	269 (64.5)
	Yes	148 (35.5)
Present chronic diseases	No	332 (79.6)
	Yes	85 (20.4)
Previous abortion	No	284 (68.1)
	Yes	133 (31.9)
Previous C-section in multiparous women (n = 262)	No	134 (51.1)
	Yes	128 (48.8)
Current pregnancy planned	No	173 (41.5)
	Yes	244 (58.5)
Health education about pregnancy	No	197 (47.2)
	Yes	220 (52.8)
Smoking	No	383 (91.8)
	Yes	34 (8.2)

**Table 3:** Results of GSES and BCFQOL-AR.

Scales	All Sample (n = 417)	Subgroup of Pregnant Women Who Do Not Have Children (n = 155)	Subgroup of Preg-Nant Women Who Have at Least One Child (n = 262)
GSES total score (m ± SD)	28.15 ± 6.016	28.30 ± 6.007	28.06 ± 6.031
GSE levels, n (%)			
Low	51(12.2)	15(9.7)	36(13.7)
Moderate	220(52.8)	86(55.5)	134(51.1)
High	146(35.0)	54(34.8)	92(35.1)
BCFQOL-AR (m ± SD)			83.90 ± 13.556
Family interaction	_	_	25.06 ± 4.659
Parenting	_	_	24.65 ± 4.849
Emotional well-being	_	_	14.23 ± 3.453
Material well-being	_	_	19.97 ± 3.736

GSES: General Self-Efficacy Scale; GSE: general self-efficacy; BCFQOL-AR: Beach Center Family Quality of Life Scale-Arabic.

**Table 4:** Univariable and multivariable linear regression analysis assessing the general self-efficacy and the associated factors among pregnant women attending antenatal care units (n = 417).

	Univariable Analysis			Multivariable Model		
Covariates	Estimate	95% CI	*p*-Value	Estimate	95% CI	*p*-Value
Age	−0.13	(−0.24, −0.03)	0.015	−0.14	(−0.14, 0.13)	0.020
Educational level						
No education		Reference			Reference	
Primary	−1.6	(−3.27, 2.95)	0.921	−0.51	(−3.42, 0.87)	0.743
Secondary	2.04	(−0.79, 4.88)	0.157	0.98		0.505
Tertiary	2.81	(−0.01, 5.63)	0.051	1.06		0.472
Marital status						
Widowed		Reference			Reference	
Married	−4.28	(−12.65, 4.09)	0.316	−3.59	(−5.86, 5.13)	0.385
Divorced	−6.50	(−15.30, 2.30)	0.147	−5.27		0.227
Employment status						
Employed		Reference			Reference	
Unemployed	−1.26	(−2.42, −0.10)	0.033	−0.44	(−2.51, 0.93)	0.477
Place of residence						
Urban		Reference				
Rural	−3.11	(−4.52, −1.70)	<0.001	−2.06		0.007
Household income level						
Moderate		Reference			Reference	
Low	−2.60	(−4.06, −1.08)	<0.001	−1.21	(−2.12, 1.56)	0.13
High	2.58	(0.07, 5.08)	0.044	2.34		0.095
Number of children						
None		Reference			Reference	
One or more	−0.24	(−1.44, 0.96)	0.695	0.72	(−4.60, 0.39)	0.342
Household size	−0.26	(−0.74, 0.22)	0.291	0.08		0.766
Has chronic diseases						
No		Reference			Reference	
Yes	−0.27	(−1.71, 1.17)	0.710	0.29	(0.03, 3.39)	0.701
Gestational age	0.09	(−0.17, 0.36)	0.490	0.16		0.233
Complications during pregnancy						
No		Reference			Reference	
Yes	−1.35	(−2.55, −0.14)	0.028	−0.88	(−3.15, 1.69)	0.161
Previous miscarriage						
No		Reference			Reference	
Yes	−0.46	(−1.70, 0.78)	0.470	0.12	(−5.37, −0.21)	0.848
Previous C-section						
No		Reference			Reference	
Yes	−0.50	(−1.76, 0.75)	0.431	0.1	(−5.35, 0.96)	0.890
Planned pregnancy						
No		Reference			Reference	
Yes	1.78	(0.62, 2.95)	0.003	1.26	(−4.01, 1.86)	0.036

**Table 5:** Univariable and multivariable linear regression analysis assessing the association between family quality of life and general self-efficacy among pregnant women with one or more children (n = 262).

	Univariable Analysis			Multivariable Model 1 *			Multivariable Model 2 **		
Covariates	Estimate	95% CI	*p*-Value	Estimate	95% CI	*p*-Value	Estimate	95% CI	*p*-Value
Age	−0.16	(−0.29, −0.02)	0.021	−0.12	(−0.27, 0.01)	0.077	−0.14	(−0.28, −0.01)	0.036
Educational level									
No education		Reference			Reference				
Primary	−0.84	(−4.49, 2.81)	0.652	−2.39	(−5.88, 1.09)	0.179	−2.22	(−5.71, 1.28)	0.214
Secondary	2.09	(−1.23, 5.42)	0.216	−0.58	(−3.87, 2.70)	0.728	−0.46	(−3.74, 2.82)	0.783
Tertiary	2.32	(−1.03, 5.68)	0.174	−0.84	(−4.21, 2.53)	0.625	−0.55	(−3.90, 2.79)	0.746
Marital status									
Married		Reference			Reference				
Divorced	−3.80	(−7.61, 0.006)	0.050	−1.89	(−5.59, 1.80)	0.316	−1.75	(−5.44, 1.94)	0.353
Employment status									
Employed		Reference			Reference				
Unemployed	−1.17	(−2.63, 0.29)	0.118	−0.16	(−1.63, 1.32)	0.834	−0.15	(−1.63, 1.33)	0.846
Place of residence									
Urban		Reference							
Rural	−2.82	(−4.61, −1.04)	0.002	−1.18	(−3.08, 0.73)	0.228	−1.19	(−3.09, 0.71)	0.219
Household income level									
Moderate		Reference			Reference				
Low	−2.99	(−4.84, −1.14)	0.001	−1.78	(−3.67, 0.09)	0.064	−1.84	(−3.71, 0.03)	0.054
High	3.10	(−0.66, 6.86)	0.105	1.33	(−2.33, 5.01)	0.476	1.55	(−2.12, 5.23)	0.408
Household size	−0.45	(−1.19, 0.29)	0.231	0.10	(−0.64, 0.84)	0.789	0.18	(−0.55, 0.91)	0.636
Has chronic diseases									
No		Reference			Reference				
Yes	−1.05	(−2.84, 0.74)	0.249	0.42	(−2.23, 1.39)	0.654	−0.04	(−1.82, 1.74)	0.968
Gestational age	0.008	(−0.34, 0.35)	0.963	0.13	(−0.19, 0.44)	0.428	0.15	(−0.16, 0.47)	0.341
Complications during pregnancy									
No		Reference			Reference				
Yes	−0.89	(−2.41, 0.63)	0.248	0.380	(−1.12, 1.88)	0.619	0.15	(−1.34, 1.64)	0.841
Previous miscarriage									
No		Reference			Reference				
Yes	−0.17	(−1.72, 1.38)	0.827	0.570	(−0.88, 2.02)	0.444	0.54	(−0.92, 1.99)	0.469
Previous C-section									
No		Reference			Reference				
Yes	−0.39	(−1.87, 1.07)	0.594	0.260	(−1.16, 1.69)	0.717	0.39	(−1.03, 1.81)	0.587
Planned pregnancy									
No		Reference			Reference				
Yes	1.44	(−0.02, 2.91)	0.053	0.684	(−0.94, 1.95)	0.495	0.46	(−0.97, 1.89)	0.529
Family interaction	0.33	(0.17, 0.48)	<0.001	−0.060	(−0.27, 0.14)	0.536		−	
Parenting	0.44	(0.30, 0.59)	<0.001	0.290	(0.09, 0.50)	0.004		−	
Emotional well-being	0.44	(0.23, 0.64)	<0.001	0.100	(−0.15, 0.36)	0.423		−	
Physical/material well-being	0.60	(0.42, 0.78)	<0.001	0.250	(0.01, 0.50)	0.043		−	
Family quality of life	0.17	(0.12, 0.22)	<0.001		−		0.15	(0.09, 0.20)	<0.001

* Model 1 used the four dimensions of the Beach Center Family Quality of Life Scale (BCFQOL) (family interaction, parenting, emotional well-being, and physical/material well-being) as exposures. ** Model 2 used only the family quality of life total score as an exposure.

## Data Availability

Data were available in the Mendeley Data Repository under this citation: Dardouri and Ayouni (2025) [50].

## Abbreviations

The following abbreviations are used in this manuscript:

GSE	General self-efficacy
FQOL	Family quality of life

## References

[1] WHO (2024). Maternal Mortality. https://www.who.int/news-room/fact-sheets/detail/maternal-mortality.

[2] WHO (2021). Why We Need to Focus on Quality Care for Women and Newborns. https://www.who.int/news/item/04-05-2021-why-we-need-to-focus-on-quality-care-for-women-and-newborns.

[3] WHO (2021). Quality of Care for Maternal, Newborn and Child Health (QoC Network). https://www.who.int/groups/Quality-of-care-network.

[4] Hajri S., Belhadj H. (2020). The role of midwives in first—trimester abortion care: A 40—year experience in Tunisia. Int. J. Gynaecol. Obstet..

[5] Amroussia N., Goicolea I., Hernandez A. (2016). Reproductive Health Policy in Tunisia. Health Hum. Rights.

[6] Strecher V.J., McEvoy DeVellis B., Becker M.H., Rosenstock I.M. (1986). The Role of Self-Efficacy in Achieving Health Behavior Change. Health Educ. Q..

[7] Schwarzer R., Luszczynska A. (1995). Self-Efficacy and Health Behavior Theories. https://cancercontrol.cancer.gov/sites/default/files/2020-06/self-efficacy.pdf.

[8] Bandura A. (1997). Self-Efficacy: The Exercise of Control.

[9] Pascual Z.N., Langaker M.D. (2023). Physiology, Pregnancy.

[10] CDC (2025). During Pregnancy. Pregnancy.

[11] DiPietro J.A. (2010). Psychological and psychophysiological considerations regarding the maternal-fetal relationship. Infant Child Dev..

[12] Ghasemi S., Rayyani M., Farokhzadian J. (2021). General health and self-efficacy for health practices of pregnant women: Is it important for motherhood?. J. Public Health.

[13] Wahyuni S., Rahayu T., Nursalam (2021). Self efficacy of pregnant women in areas affected by COVID-19. Enferm. Clínica.

[14] Ma R., Yang F., Zhang L., Sznajder K.K., Zou C., Jia Y., Cui C., Zhang W., Zhang W., Zou N. (2021). Resilience mediates the effect of self-efficacy on symptoms of prenatal anxiety among pregnant women: A nationwide smartphone cross-sectional study in China. BMC Pregnancy Childbirth.

[15] Campbell T.L., McDaniel S.H., Cole-Kelly K., Paulman P., Taylor R.B., Paulman A.A., Nasir L.S. (2020). Family Issues in Health Care. Family Medicine: Principles and Practice.

[16] Luo Z.N., Li K., Chen A.Q., Qiu Y.C., Yang X.X., Lin Z.W., Liu J.H., Wu Y.B., Chen J.Y. (2024). The influence of family health on self-efficacy in patients with chronic diseases: The mediating role of perceived social support and the moderating role of health literacy. BMC Public Health.

[17] Bahari G., Scafide K., Krall J., Mallinson R.K., Weinstein A.A. (2019). Mediating role of self-efficacy in the relationship between family social support and hypertension self-care behaviours: A cross-sectional study of Saudi men with hypertension. Int. J. Nurs. Pract..

[18] Chang C.-W., Chen F. (2022). Family social networks and personal recovery among Chinese people with mental illness in Hong Kong: The mediating effects of self-esteem and self-efficacy. Psychiatr. Rehabil. J..

[19] Wensu Z., Xidi Z., Shaojie L., Baohua Z., Yunhan Y., Huilan X., Zhao H., Xiyue X. (2021). Does the Presence of Anxiety and Depression Symptoms Mediate the Association Between Family Functions and Self-Efficacy in Pregnant Women in the Third Trimester?: A Community-Based Cross-Sectional Survey. Front. Psychiatry.

[20] Zakiei A., Norouzi E., Ghasemi S.R., Komasi S., Rostampour M., Khazaie H. (2022). Controlling risky behavior associated with AIDS: The role of social support, family functioning, self-efficacy and AIDS risk perception. BMC Psychol..

[21] Puspasari J., Nur Rachmawati I., Budiati T. (2018). Family support and maternal self-efficacy of adolescent mothers. Enferm. Clínica.

[22] Francisco Mora C., Ibáñez A., Balcells-Balcells A. (2020). State of the Art of Family Quality of Life in Early Care and Disability: A Systematic Review. Int. J. Environ. Res. Public Health.

[23] Poston D., Turnbull A., Park J., Mannan H., Marquis J., Wang M. (2003). Family quality of life: A qualitative inquiry. Ment. Retard..

[24] Alnahdi G.H. (2022). Rasch validation of the Arabic version of the beach center family quality of life scale (BCFQOL-AR). Front. Psychol..

[25] Zheng B., Yu Y., Zhu X., Hu Z., Zhou W., Yin S., Xu H. (2020). Association between family functions and antenatal depression symptoms: A cross-sectional study among pregnant women in urban communities of Hengyang city, China. BMJ Open.

[26] Frini O., Muller C. (2023). Revisiting fertility regulation and family ties in Tunisia. BMC Pregnancy Childbirth.

[27] Ayoub L. (2025). Disparity of the Health Sector in Tunisia in Favor of the Centre-Eastern Region, the Sahel and Sousse Governorate. Int. J. Sci. Technol..

[28] Dhand N.K., Khatkar M.S. (2014). Statulator: An Online Statistical Calculator. Sample Size Calculator for Estimating a Single Proportion. https://scholar.google.com/citations?user=FYipWOUAAAAJ&hl=en.

[29] Crandall A., Rahim H.F.A., Yount K.M. (2016). Validation of the General Self-Efficacy Scale among Qatari young women. East Mediterr. Health J..

[30] Schwarzer R., Jerusalem M., Weinman J., Wright S. (1995). Generalized Self-Efficacy Scale. Measures in Health Psychology: A User’s Portfolio. Causal and Control Beliefs.

[31] Kärner Köhler A., Tingström P., Jaarsma T., Nilsson S. (2018). Patient empowerment and general self-efficacy in patients with coronary heart disease: A cross-sectional study. BMC Fam. Pract..

[32] Bień A., Grzesik-Gąsior J., Ćwiek D., Pięta B., Kulesza-Brończyk B., Bączek G. (2025). Self-efficacy as a moderator in the relationship between body image and health behaviors in pregnant women. Sci. Rep..

[33] Cui C., Zhai L., Sznajder K.K., Wang J., Sun X., Wang X., Zhang W., Yang F., Yang X. (2021). Prenatal anxiety and the associated factors among Chinese pregnant women during the COVID-19 pandemic—A smartphone questionnaire survey study. BMC Psychiatry.

[34] Silva-Tubio J.R., Oliver-Roig A., Perpiñá-Galvañ J., Richart-Martínez M. (2021). Reliability and validity of the reduced Spanish version of the Prenatal Breastfeeding Self-Efficacy Scale. Res. Nurs. Health.

[35] Hoffman L., Marquis J., Poston D., Summers J.A., Turnbull A. (2006). Assessing Family Outcomes: Psychometric Evaluation of the Beach Center Family Quality of Life Scale. J. Marriage Fam..

[36] Brown I., Brown R.I., Baum N.T., Isaacs B.J., Myerscough T., Neikrug S. (2006). Family Quality of Life Survey Main Caregivers of People with Intellectual or Developmental Disabilities.

[37] Alnahdi G.H., Alwadei A., Woltran F., Schwab S. (2022). Measuring Family Quality of Life: Scoping Review of the Available Scales and Future Directions. Int. J. Environ. Res. Public Health.

[38] Alnahdi G.H., Schwab S., Elhadi A. (2021). Psychometric Properties of the Beach Center Family Quality of Life Scale: Arabic Version. J. Child Fam. Stud..

[39] Rahaju S., Lucas G.G.G., Putri S.D.A. (2024). Internal Structure of the Beach Center Family Quality of Life Scale: Indonesian Version. J. Educ. Health Community Psychol..

[40] Risnawaty W., Agustina Suryadi D. (2020). Psychometric Properties of Beach Center Family Quality of Life Scale for Indonesian Families’ Children Without Disabilities. Proceedings of the 2nd Tarumanagara International Conference on Applied Social Science and Humanities (TICASH 2020).

[41] Schwarzer R., Renner B. (2000). Social-cognitive predictors of health behavior: Action self-efficacy and coping self-efficacy. Health Psychol..

[42] WageIndicator Foundation (2025). Minimum Wage in Tunisia. WageIndicator Foundation. https://wageindicator.org/salary/minimum-wage/tunisia.

[43] Simon T., Fikadu K., Afework B., Alemu H., Kussia B. (2024). Childbirth Self-Efficacy and Its Associated Factors among Pregnant Women in Arba Minch Town, Southern Ethiopia, 2023: A Cross-Sectional Study. J. Pregnancy.

[44] Elbeji A., Mezlini I. (2020). Many Tunisians Lack Adequate Access to Health Care and Water, Rate Government Poorly. https://www.afrobarometer.org/publication/ad393-many-tunisians-lack-adequate-access-health-care-and-water-rate-government-poorly/.

[45] UNICEF Middle East and North Africa (2024). Tunisia Country Brief. Community Health: Policy and Implementation Landscape Mapping in the Middle East and North Africa Region 2024. https://www.unicef.org/mena/reports/tunisia-country-brief.

[46] Trahar S., Timmis S., Lucas L., Naidoo K. (2020). Rurality and access to higher education. Comp. J. Comp. Int. Educ..

[47] Agyekum B. (2023). Adult unmet educational needs: Higher education options amongst adults in rural and small towns in Ghana. J. Adult Contin. Educ..

[48] Soh Y.X., Razak N.K.B.A., Cheng L.J., Lau Y. (2020). Determinants of childbirth self-efficacy among multi-ethnic pregnant women in Singapore: A structural equation modelling approach. Midwifery.

[49] Zhang L., Ren L., Li H., Qiu H., Yang H., Shi X., Rong H., Zheng C., Li F., Luo T. (2025). The effects of maternal health literacy, family functioning and self-efficacy on antepartum depression in pregnant women in China: A moderated mediation model. BMC Psychiatry.

[50] Dardouri M., Ayouni I., Maternal Mental Health (2025). Mendeley Data, V1. https://data.mendeley.com/datasets/zsmb2xsdmx/1.

